# Passage-dependent accumulation of somatic mutations in mesenchymal stromal cells during *in vitro* culture revealed by whole genome sequencing

**DOI:** 10.1038/s41598-017-15155-5

**Published:** 2017-11-06

**Authors:** Myungshin Kim, Je-Keun Rhee, Hayoung Choi, Ahlm Kwon, Jiyeon Kim, Gun Dong Lee, Dong Wook Jekarl, Seungok Lee, Yonggoo Kim, Tae-Min Kim

**Affiliations:** 1 Department of Laboratory Medicine, College of Medicine, The Catholic University of Korea, Seoul, Republic of Korea; 2 Catholic Genetic Laboratory Center, Seoul St. Mary’s Hospital, College of Medicine, The Catholic University of Korea, Seoul, Republic of Korea; 3 Cancer Research Institute, College of Medicine, The Catholic University of Korea, Seoul, Republic of Korea; 4 Department of Medical Informatics, College of Medicine, The Catholic University of Korea, Seoul, Republic of Korea

## Abstract

Human mesenchymal stromal cells (MSCs) have served as a major cellular resource for cell-based immunomodulatory and regenerative therapies. However, genomic instability may accumulate during *ex vivo* expansion of MSCs, thereby increasing the potential of malignant transformation. Here, we performed whole genome sequencing of two peripheral blood-derived MSC lines (MSC1 and MSC2) at various passages (passage 1 [P1] to P9). The majority of single-nucleotide variations (SNVs) occurred in later passages; specifically, 90% and 70% of all SNVs in MSC1 and MSC2 were observed in P9 and P7/P9, respectively. These late-occurring SNVs were enriched with C > A transversions and were overrepresented in intronic regions compared to intergenic regions, suggesting that the mutational forces are not constant across the passages. Clonality analyses also distinguished early-occurring, subclonal SNVs from late-occurring, clonally fixed SNVs. In addition, MSCs were largely devoid of copy number alterations (CNAs) (i.e., 0–2 CNAs per passage), with one exception (MSC2-P3) harboring 29 passage-specific CNAs. Our findings suggest that the SNVs found to be abundant at later passages likely resulted from the accumulation of replication stress, which can be associated with proliferation activity. Thus, the genomic instability associated with proliferation records should be considered for clinical applications of MSCs.

## Introduction

Since the discovery of human mesenchymal stromal cells (MSCs) in the bone marrow^1^, MSCs have served as a valuable resource for cell-based therapeutics. Current clinical trials of MSC-based therapies for degenerative diseases have shown promising results^2,3^, and the applications of MSCs have been expanded into immunomodulation to treat graft-versus-host disease and other immune diseases^4^. MSCs can be obtained from various sources such as bone marrow, adipose tissues, umbilical cords, and peripheral blood^5,6^. Due to the rarity of MSCs in these biological sources, however, *ex vivo* expansion is usually required to achieve the desired cell numbers for *in vivo* use. One of the major concerns associated with such long-term *in vitro* culture of MSCs is genomic instability. We define genomic instability of MSCs as the abundance of any type of genomic alterations somatically acquired during the establishment and maintenance of MSCs. Although the majority of all randomly acquired somatic mutations are expected to be functionally neutral, some of these mutations might be responsible for cellular senescence or malignant transformation during *ex vivo* expansion or future *in vivo* use.

The genomic instability of MSCs has been predominantly investigated using microarray-based tools such as array-based comparative genomic hybridization (array-CGH) to identify chromosomal copy number alterations (CNAs) such as chromosomal gains and losses. These studies have reported that, although cultured MSCs are largely devoid of large-scale CNAs^7^, late-passage MSCs may harbor a substantial number of CNAs^8^. Recent technological innovations such as whole genome sequencing have enabled the genomic characterization of various types of genomic alterations at high resolution. While next generation whole genome and whole exome sequencing have been adapted for other types of human stem cells such as induced pluripotent stem cells^9–11^, only a few studies have applied these technologies to MSCs. In one of these studies, Bhutani *et al*. performed whole genome sequencing of a single MSC line at multiple passages (passage 1 [P1], P8, and P13) and revealed the dynamics of single-nucleotide substitutions (SNVs) as well as the relative absence of CNAs^12^.

In this study, we investigated the genomic instability of two MSC lines during *in vitro* culture on a genome-wide scale at high resolution using whole genome sequencing. Three types of genomic alterations - SNV, short insertion/deletion (indel), and CNA - were investigated across multiple passages (P1, P3, P5, P7, and P9) to reveal the mutation abundances and clonal origins of SNVs during *ex vivo* expansion. In addition, we characterized the mutation dynamics of MSCs by comparing mutations across different passages of given individuals.

## Results

### Growth kinetics of MSCs during *in vitro* culture

**Figure 1:**
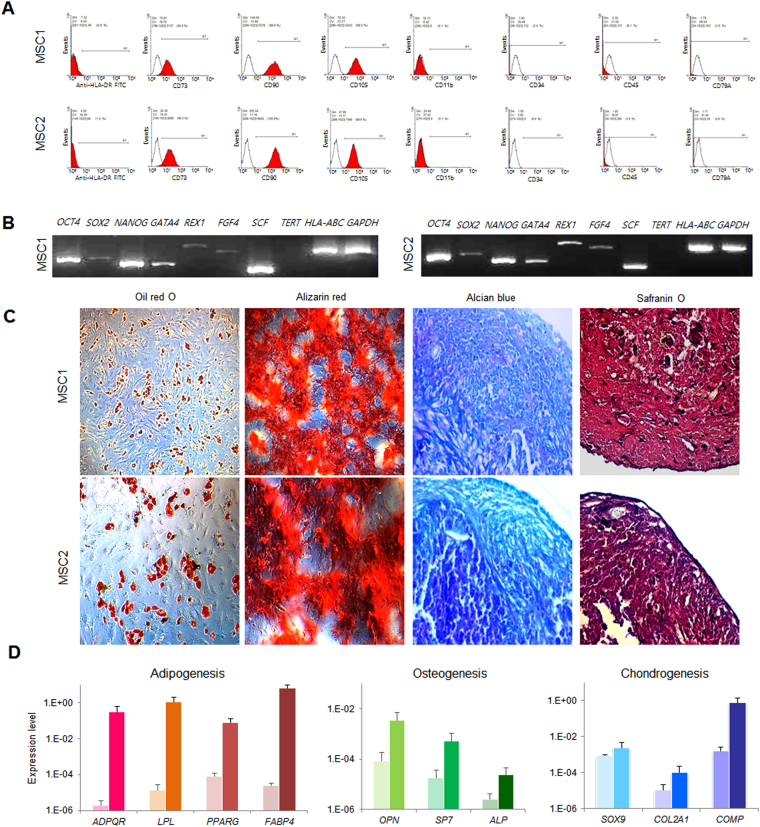
Characteristics of peripheral blood-derived MSCs. (**A**) Immunophenotype analyses of using flow cytometry. Red histograms represent antibody staining and black histograms indicate isotype matched IgG controls. (**B**) Gene expression of stemness markers by RT-PCR. (**C**) Evaluation of mesodermal differentiation potential in terms of adipogenesis (Oil red O), osteogenesis (Alizarin red) and chondrogenesis (Alcian blue and Safranin O) (×100). (**D**) qRT-PCR of native (d0, left) and differentiated (d14, right) cells presenting the expression of genes associated with trilineage differentiation. The results are represented as mean and standard deviation from three independent experiments.

We cultured two MSC lines (MSC1 and MSC2) from the peripheral blood mononuclear cells (PBMCs) of two different individuals. The adherent PBMCs displayed a fibroblast-like morphology after 2 weeks of culture. They also presented a typical immunophenotype of MSC; specifically, >95% positive rates for MSC-specific markers of *CD105*, *CD90*, and *CD73* and <2% positive rates for hematopoietic stem cell markers of *CD45*, *CD34*, *CD79a*, *CD11b*, and *HLA-DR* (Fig. 1A). The MSCs also expressed stemness-related genes such as *OCT4*, *SOX2*, *NANOG*, *GATA4*, *REX1*, *FGF4*, *SCF*, and *HLA*-*ABC*, whereas they did not express *TERT* (Fig. 1B). The *TERT* gene encodes one component of telomerase, which maintains the length of telomeres. Previous study demonstrated that telomerase activity was low in human MSCs^13^ and became undetectable during *in vitro* culture^8^. The MSC lines (P3) possessed trilineage differentiation potential which was demonstrated by morphology with special staining and gene expression with quantitative reverse transcription polymerase chain reaction (qRT-PCR) (Fig. 1C,D).

**Figure 2:**
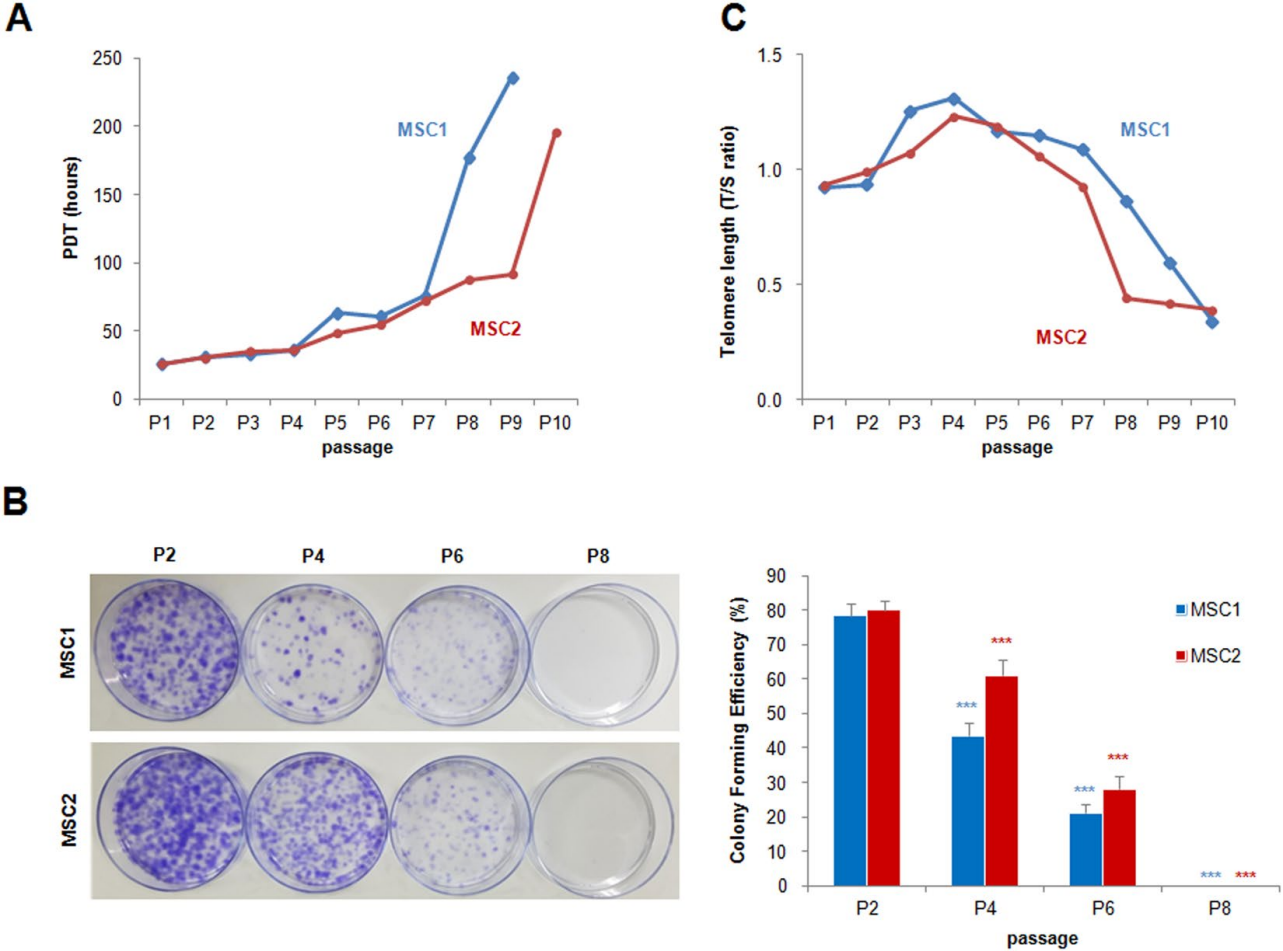
Growth kinetics of peripheral blood-derived MSCs. (**A**) MSCs during passaging. Data from two donors are presented by plotting population doubling time (PDT; *y*-axis) against passages (*x*-axis). (**B**) Colony-forming unit-fibroblast (CFU-F) assays of MSCs. Comparison of CFU-Fs from early to late passage cultures. The results are represented as mean and standard deviation from five independent experiments. ***P < 0.001. (**C**) Relative telomere length decreasing shown as T/S ratios (T, telomere; S, single-copy gene) during passaging.

The growth kinetics of MSC cultures were assessed by calculating their population doubling times (PDTs) and colony-forming unit-fibroblast (CFU-F) assay. The PDTs of MSC1 and MSC2 were both less than 40 hours until P4; at P5, the PDT was slightly increased (63.31 and 48.09 hours, respectively). The PDT was markedly increased at P8 in MSC1 (176.7 hours) and P10 in MSC2 (195.1 hours). MSC1 and MSC2 stopped proliferating at P9 and P10, respectively (Fig. 2A). As expected, the number of CFU-Fs declined for the cells with high passage numbers (Fig. 2B). Along with the impaired proliferation, replicative senescence of MSCs was determined by the cellular morphology and telomere length. Enlarged granular cells were gradually increased during *in vitro* subculture and debris was formed in the medium. The granular cell began to be vacuolated and finally detached from the base of the flasks. We measured telomere length using qPCR^14^ and obtained the relative telomere to single copy gene (T/S) ratio. The range of the T/S ratio varied between 0.34 to 1.31 in different MSCs and in different passages. The T/S ratio decreased after P7, suggestive of a telomere shortening during cellular expansion (Fig. 2C).

### Somatic gene alterations acquired during ***in vitro*** culture of MSCs

Whole genome sequencing was performed across various passages during *ex vivo* expansion, ranging from the initial establishment (passage 1 or [P1]) to multiple subsequent passages (P3, P5, P7, and P9). Whole genome sequencing was also performed on blood mononuclear cells to obtain matched normal genomes. The information of whole genome sequencing data, including sequencing depth, is available in Supplementary Table S1. By comparing the sequencing data of MSCs with those of their matched normal controls, we identified three types of somatic alterations as major categories of genomic instability: SNV, indel, and CNA. We investigated the abundance of each of these three types of genomic alterations and their clonal patterns with respect to passage.

**Figure 3:**
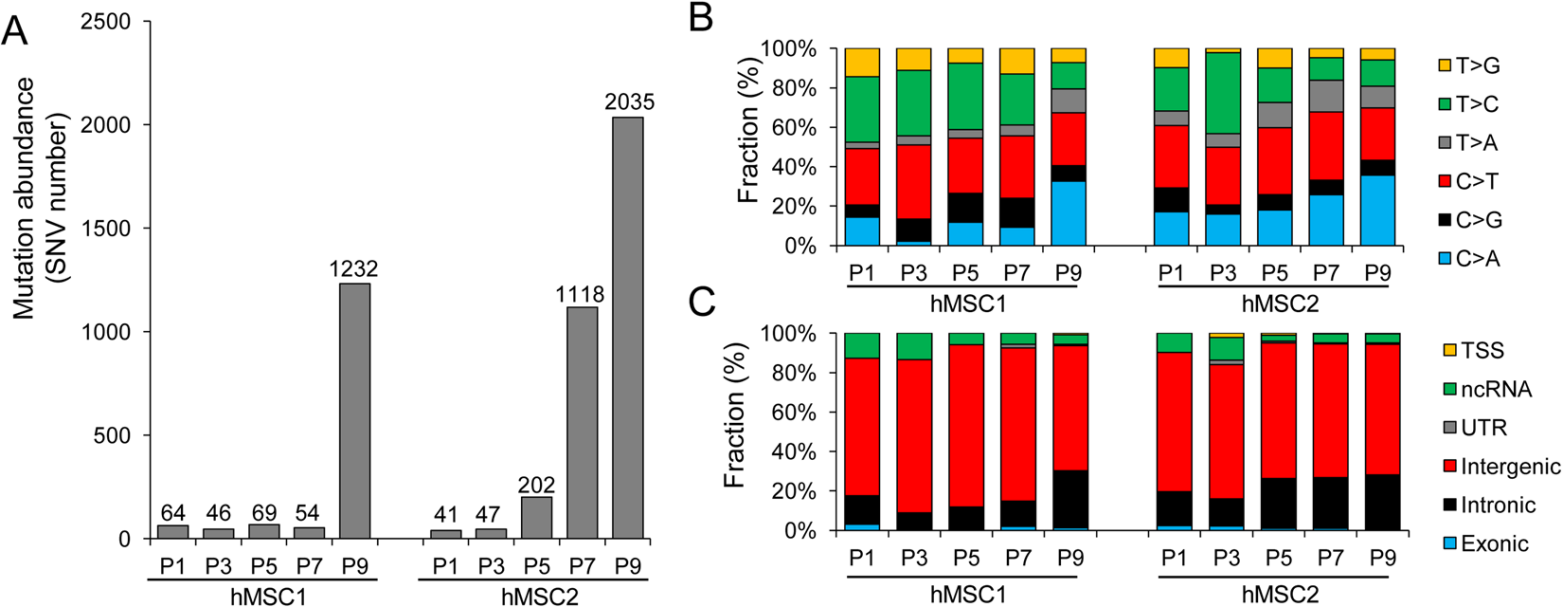
Abundances and spectra of somatic mutations acquired during *ex vivo* expansion of MSCs. (**A**) Mutational abundances (i.e., numbers of SNVs) are shown across P1 to P9 for the two MSC lines (MSC1 and MSC2) examined. The numbers of SNVs are shown above each bar. The SNVs were identified by comparing MSCs at each passage with their matched control cells. (**B**) The six categories of mutation spectra with respect to sequence change are shown as fractions (%) of the given somatic SNVs across the passages. (**C**) The functional categories or location of SNVs with respect to nearby genes are shown. Exonic mutations include missense (nonsynonymous), silent (synonymous), nonsilent, and splicing mutations. TSS mutations are those residing upstream or downstream of the transcription start sites.

Among the somatic mutations representing MSC genomic instability, we first investigated the mutational abundance of SNVs (Fig. 3A). Of particular note, we observed that both MSC1 and MSC2 showed an abrupt increase in SNV abundance in later passages, i.e., 84.0% and 91.6% of all SNVs in MSC1 and MSC2 were observed at P9 and P7-P9, respectively. The full list of somatic SNVs is available in Supplementary Table S2. We also investigated the mutation spectra and sequence changes of somatic SNVs across the passages (Fig. 3B). We observed that C > A transversions were enriched and coincided with the increase of mutation abundance in later passages. In addition, analysis of SNV location with respect to nearby genes revealed that intronic SNVs were enriched than intergenic SNVs in later passages (Fig. 3C). These findings suggest that SNVs occur abundantly at later passages and also show distinct mutational features, indicating that the mutational forces that operate during the *ex vivo* expansion of MSCs are not constant across passages.

Out of all the identified SNVs, 33 occurred in coding sequences. We further investigated the functional consequences of these SNVs (Supplementary Table S3). Truncating (i.e., functionally inactivating) nonsense mutations were observed in *OTUD5* (OTU deubiquitinase 5), which encodes a protein with putative deubiquitinase activity associated with the innate immune and p53 pathways^15,16^. However, the roles of these mutations in stem cell maintenance and tumorigenicity are unclear, along with the effect of the nonsense mutation in *LOC440243*. In addition to the five silent or synonymous SNVs, we identified 26 missense or nonsynonymous SNVs. None of the 33 coding mutations were observed in any of the 595 genes listed in the Cancer Gene Census that were reported to have a potential oncogenic role^17^. In addition, the observed missense mutations were not present in any of the missense mutation hotspots listed in the COSMIC database (<5 occurrences out of the 27 missense mutations)^18^. Although our results require further validation, they suggest that the missense mutations acquired during the *ex vivo* expansions are largely functionally neutral passengers.

**Figure 4:**
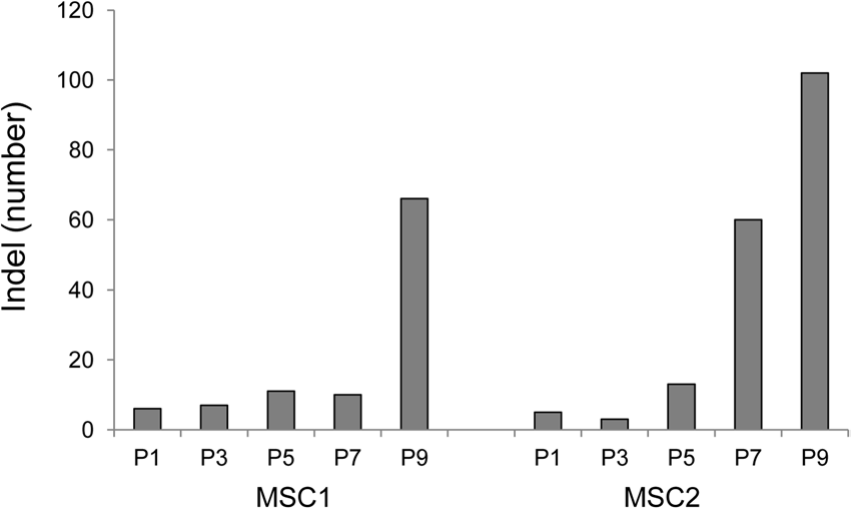
The abundance of indels acquired during *ex vivo* expansion of MSCs. Indel abundances (i.e., numbers of indels) are shown across passages (P1 to P9).

We also identified 283 indels that accumulated throughout the passages of the two MSC lines. The list of indels is available in Supplementary Table S4. The indel abundance patterns were similar to those of the SNVs, i.e., the indel frequencies increased abruptly at P9 and P7-P9 for MSC1 and MSC2, respectively (Fig. 4), suggesting that similar mutational forces drove the accumulation of both SNVs and indels. No coding indels were observed, also suggesting that these mutations were not likely to be functional events.

### Mutational dynamics across the passages

**Figure 5:**
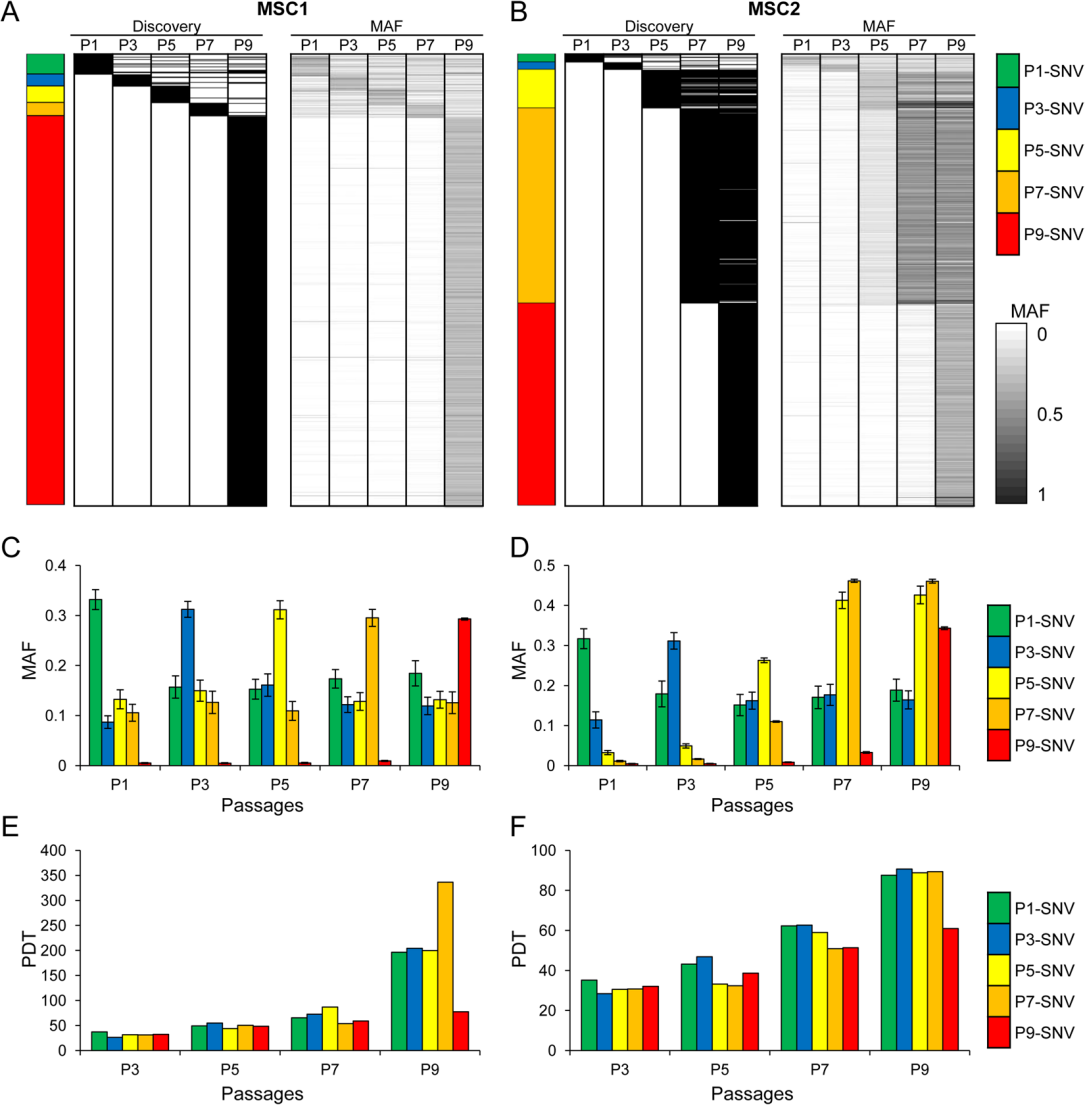
Clonal analyses of mutations. (**A**) Each row (horizontal line) represents an individual SNV identified in MSC1. The two heatmaps show the passage at which the corresponding mutations were identified (“Discovery”; left) and the MAF or mutation allele frequencies across the passages (“MAF”; right), respectively. A colored bar (left) indicates the passage assigned to the individual SNVs. If a single mutation was identified at multiple passages, the mutation was assigned to the earliest passage. Mutations of P1 (green), P3 (blue), P5 (yellow), P7 (orange), and P9 (red) are distinguished with respective colors. (**B**) The mutations identified in MSC2 are similarly shown. (**C**) Mean of MAFs are plotted for five mutation classes (P1–P9 with respective colors) across five passages of MSC1. Error bars represent the standard error. Except for the P9 mutations (red) whose MAF abruptly increased at P9, P1-P7 mutations show a fluctuation of MAFs across the passages. (**D**) Similarly shown for the MAF of five mutation classes (mean values with standard error) of MSC2. (**E** and **F**) Mean of simulated values of the PDT are plotted for five mutation classes of MSC1 (**E**) and MSC2 (**F**).

To investigate the evolutionary history of the somatic mutations (e.g., at which passage each mutation arose and whether the mutation was clonally fixed or swept away in subsequent passages), we examined the mutation allele frequency (MAF) of each somatic mutation. The passage at which each SNV was identified and the MAF distributions across the passages are shown in Fig. 5 (“Discovery” and “MAF” panels, respectively). To account for redundancy, we defined P1-SNVs as those first identified at P1 by a mutation-calling algorithm across the passages. Thus, P1-SNVs (e.g., green in Fig. 5A) comprised those solely identified at P1 and also those identified at P1 and other passages. P3-, P5-, P7-, and P9-SNVs were categorized similarly (blue, yellow, orange, and red, respectively).

For MSC1, the SNVs identified at P1 to P7 (P1- to P7-SNVs) were distinguished from the P9-SNVs (Fig. 5A). Specifically, the P1- to P7-SNVs had the highest MAFs at their corresponding passages (e.g., the MAF of P1-SNVs was highest at P1). However, these MAFs were also present at lower frequencies in the other passages (Fig. 5A,C), and these mutations were often identified at more than one passage. These findings suggest that the mutations identified at early passages (or early-occurring mutations) may be present at low frequency during *ex vivo* expansion. In addition, given that these mutations showed low MAFs from as early as P1, these mutations appear to represent low-frequency mutations acquired before or during the establishment (P1) of the MSC line. In contrast, the P9-SNVs of MSC1 were mostly absent in the preceding passages (Fig. 5A), as shown in the mean MAF plot of P9-SNVs (red; Fig. 5C). This finding suggests that these SNVs represent largely *de novo* mutations arising at later passages during *ex vivo* expansion.

For MSC2, the P1- and P3-SNVs showed MAF patterns similar to those of the MSC1 P1-P7 SNVs. Specifically, the SNVs showed elevated MAFs at P1 or P3, but were also present at lower frequencies across the passages (Fig. 5B). Of note, more than half of the SNVs in MSC2 were identified at P5 or P7; these SNVs also became clonally fixed in the subsequent passages. The MAF plots further show that the P7-SNVs showed a substantial level of MAF at P5, suggesting that the P5- and P7-SNVs originated at P5 (Fig. 5D). The MSC2 P9-SNVs showed similar MAF profiles to the MSC1 P9-SNVs, suggesting that these mutations are mostly *de novo* mutations acquired at P7 or P9.

We further simulated the PDTs of SNVs to evaluate the amplification capacity of MSCs with P1- to P9-SNVs (Fig. 5E,F). The estimated counts of MSCs with SNVs were calculated via multiplying the expanded cell number by MAF at each passage. The amplification capacity of each SNV was estimated as the harvested cell count divided by the initial seeded cell count.

The PDTs increased during passaging, meaning that all MSCs would be likely to die after repeated subculture. However, MSCs harboring P9-SNVs exhibited lower simulated PDTs than MSCs with other mutations or without mutations. The number of gained mutations was associated with the amplification power of MSCs, as evidenced by the higher numbers of P5-, P7-, and P9-SNVs in MSC2 with greater amplification compared to those of MSC1. In addition, the final expanded cell number at P9 of MSC2 was 624 times higher than that of MSC1.

### Copy number alterations

**Figure 6:**
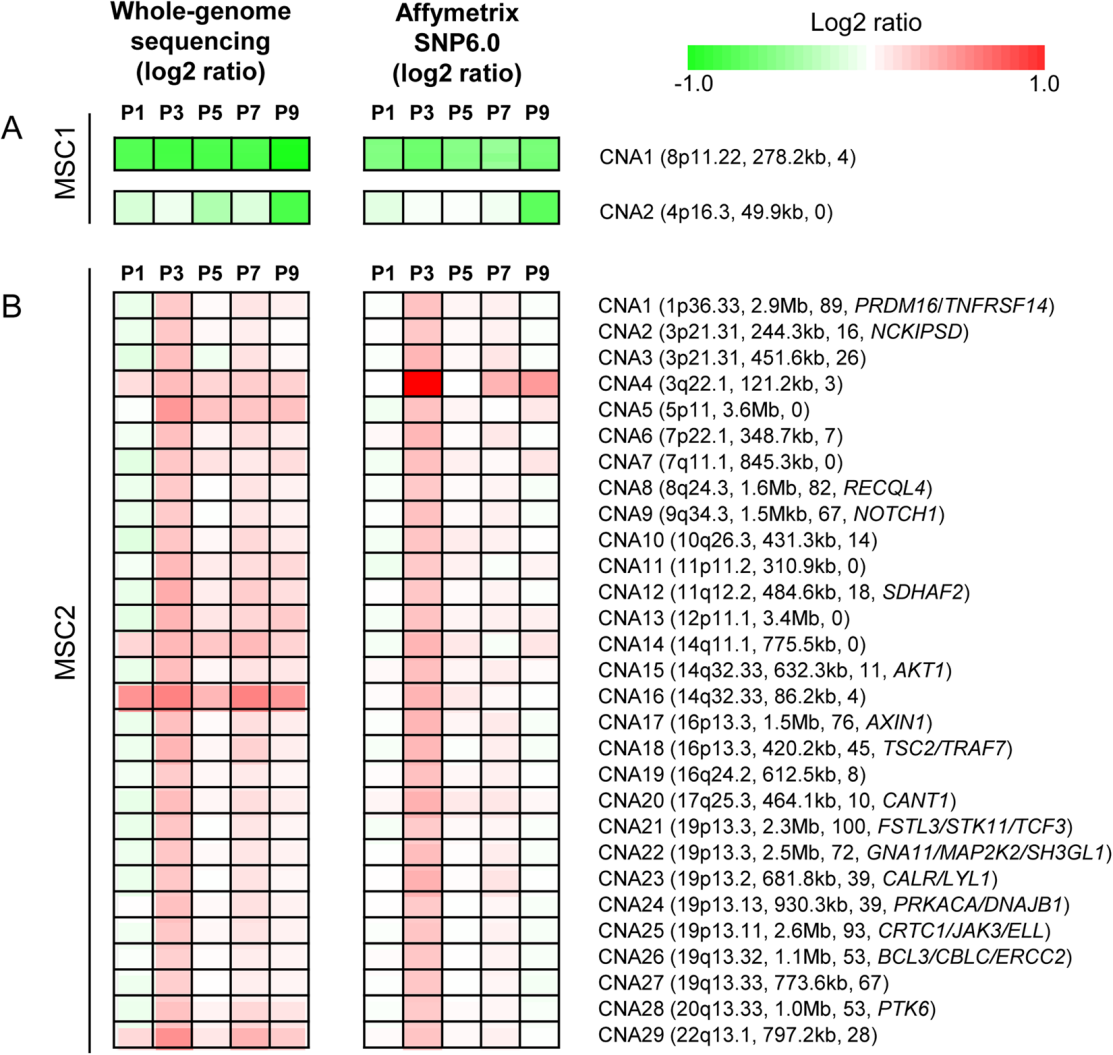
CNA segments of MSCs. (**A**) Log_2_ copy number ratios for the two CNAs identified in MSC1 by whole genome sequencing (left) and Affymetrix SNP6.0 (right) across P1 to P9 are shown. Red and green represent copy number gains and losses, respectively, as shown in the color indicator on the right. For the two CNAs, the corresponding cytobands, segment sizes, and number of genes (included in parentheses) are also shown. If the segments included known cancer-related genes from the Cancer Gene Census database, the symbols are also shown. (**B**) A similar plot for the 29 CNAs identified in MSC2-P3.

To identify CNAs, we performed microarray-based genotyping using the Affymetrix SNP6.0 platform. We jointly analyzed the microarray-based CNA calls with those from whole genome sequencing to obtain high-confidence CNAs. The CNAs identified in MSC1 and MSC2 are illustrated in Fig. 6A,B, respectively. Only two CNAs were observed for MSC1 (CNA1 and CNA2); one of these was clonal and the other subclonal. These CNAs were found at chromosomes 8p11 and 4p16, respectively (Fig. 6A). Our findings suggest that relatively few CNAs were present. Examination of log ratios revealed that MSC1-CNA1 exhibited constant copy number losses across passages, which is evidence of clonally fixed copy number losses. Although it is not clear whether MSC1-CNA2 was acquired *de novo* at P9 or whether low-frequency MSC1-CNA2 were present in earlier passages, our data demonstrate that MSC1-CNA2 had a marked copy number loss at P9.

Of particular note, 29 CNAs were observed in MSC2 at P3 (Fig. 6B). The identified CNAs varied widely in segment size (86.2 kb to 3.65 Mb; median of 77.3 kb) and number of genes (0 to 100 genes; median of 26 genes). Some of the genes belonging to these segments are known to be cancer-related. The log_2_ ratios of the segments identified across the passages show that these CNAs were acquired largely *de novo* at P3 and may have been present at low frequencies in the subsequent passages. The acquisition of a substantial number of CNAs at a particular passage, as shown in Fig. 6B, suggests that a certain cell in the population may have experienced massive genomic alterations as part of a punctuated genome evolution. As shown in Fig. 6B, the cells that acquired CNAs may have populated P3. However, our data suggest that the corresponding clones were replaced by other clones harboring substantial numbers of SNVs.

## Discussion

MSC-based cell therapies have shown promising results in various clinical fields, including regenerative medicine and immunomodulatory therapy. Compared to iPS cells (induced pluripotential stem cells) and ESCs (embryonic stem cells), MSCs are considered to be relatively safe in terms of genomic instability. However, the extent of MSC genomic instability has not yet been thoroughly investigated using high-resolution genome-wide techniques. The only whole genome sequencing study performed to date, to the best of our knowledge, analyzed one MSC cell line with limited passage information^12^. Our study of two MSC lines, which collected whole genome sequencing data from a range of passages provides valuable insights into the genomic instability in terms of the mutational abundances and dynamics during *ex vivo* culture.

For SNVs, early-occurring SNVs showed elevated MAFs at the passages at which they were identified, but to a lesser extent across the other passages. This finding suggests that the subclones harboring these mutations were subjected to genetic drift, and that these variants are likely to be functionally neutral. Somatic SNVs acquired during *ex vivo* expansion have been proposed to be present at low frequency in the peripheral blood^12^. Although the origins of these early-occurring SNVs should be further investigated, our results demonstrate that these early-occurring SNVs may have been present at low frequency prior to their detection and were not subject to clonal selection upon extended expansion.

We observed that the two MSC lines showed different features in terms of mutational burst in late passages. Specifically, MSC1 showed a P9-specific mutational burst, whereas MSC2 showed at least two waves of mutational bursts (P5 and P9). Since P9 was the last passage examined, it is unclear whether the P9-SNVs were clonally fixed in subsequent passages. However, given that the MSC2-P5/-P7 SNVs were acquired *de novo* and clonally fixed, it is likely that the MSC1 and MSC2 P9-SNVs resulted from genomic instability that favored SNVs and indels, the majority of which were fixed in the *ex vivo* expanded cell population. We were not able to identify the potential functional relevance of the coding SNVs identified because the MSCs ceased cleavage at those passages. The late-occurring MSC SNVs are worthy of further investigation; specifically, the abundance of these SNVs merits further investigation, as well as the findings that the clones harboring those mutations escaped or delayed replicative senescence. Of note, the PDT simulation data demonstrated that the MSCs that accumulated late-occurring SNVs *de novo* exhibited delayed replicative senescence rather than increasing proliferation compared to MSCs with other mutations or without mutations. It is also relevant that the cells with better proliferation capacity acquired more *de novo* mutations at late passages, such that clones harboring the mutations seemed to dominate in the population. The interindividual differences of late-occurring SNVs also support this conclusion since MSC2, which maintained higher proliferation at later passages, gained more *de novo* mutations compared to MSC1 after P5.

A recent sequencing-based analysis on human pluripotent, embryonic stem cells identified recurrent, cancer-relevant *TP53* mutations that may confer selective advantages of the affected clones^19^. This report as well as our findings highlight the need for careful examination of the cells for the presence of potentially harmful, cancer-related genomic aberrations. In addition, it has been reported that clinical grade MSCs demonstrated a decreased activity of colony formation and also a low level of DNA damage after subculture^20^. Thus, it will require further investigation whether the clinical grade MSCs cultured under good manufacturing practice (GMP) conditions show distinct mutation profiles in quantity and quality compared to our cells under standard laboratory conditions.

The relatively low numbers of CNAs in our cases, except for MSC2-P3, is consistent with previous reports. However, the unexpected abundance of CNAs in MSC2-P3 is notable. The observed CNAs were mostly acquired *de novo* at P3 and present at low frequency in subsequent passages. We conclude that genomic instability triggering CNAs occurred in MSC2-P3, but that these alterations did not confer survival benefits. We hypothesize that CNAs occurring during *ex vivo* expansion of MSCs may not be functionally advantageous for the affected clones. Compared to SNVs, CNAs may be deleterious, and the subclones harboring CNAs may be absent, or present as rare populations, in *in vitro* cultured MSCs.

In summary, MSCs exhibited genomic instability during *ex vivo* expansion. The largest change was the SNVs observed at later passages. These SNVs were enriched with C > A transversions and overrepresented in intronic regions compared to intergenic regions, while CNAs were relatively rare. Our findings suggest that early-occurring SNVs and CNAs are likely to be subject to clonal sweeps or genetic drift, but that late-occurring SNVs are abundant and clonally fixed. The SNVs abundant at later passages likely resulted from the accumulation of replication stress, which is associated with proliferation activity. Based on these results, we suggest that genomic instability, which is represented by mutational abundance, can be predicted by proliferation history. In addition, the interpretation of mutation profiles obtained from two individuals requires a caution due to a small cohort size. More samples will be required to obtain robust estimates on the abundance of somatic mutations and CNAs with respect to passage numbers as well as their confidence intervals. It is also worthy of further investigation to understand the potential impact of germline genetic makeup or other clinical features of individuals on the mutation abundance or genomic instability of MSC.

## Materials and Methods

### MSC culture and characterization

PBMCs were obtained by mobilizing the peripheral blood of two healthy hematopoietic stem cell donors. Donor1 was 15 year-old female and donor 2 was 27 year-old male. Granulocyte colony-stimulating factor (G-CSF) was administered to donors (10 μg/kg/day) by subcutaneous injection and continued for 5 days. Leukaphereses were performed on day 5 of G-CSF administration. To isolate of MSCs, 200 μL of PBMCs was mixed with 10 mL RPMI medium (Gibco, NY, USA) containing 15% (v/v) fetal bovine serum (FBS; Gibco), 1% (v/v) glutamax™-I (Gibco), and 1% (v/v) penicillin-streptomycin (10,000 U/mL, Gibco). Cells were then cultured in a T-25 culture flask at 37 °C with 5% CO_2_. After 5–6 days, adherent cells were visible on the bottom of the flask, and we replaced media with α-modified minimum essential medium (α-MEM; Gibco) containing 15% (v/v) FBS, 1% glutamax™-I (v/v), and 1% penicillin-streptomycin (10,000 U/mL, v/v). After 2–3 days, cells were harvested using 0.25% (w/v) trypsin-EDTA (Gibco) and replated all harvested cells in 150 mm culture dishes. MSCs were cultured and harvested at 70–80% confluency by treatment with 0.25% (w/v) trypsin-EDTA. The harvested cells were counted using a C-Chip hemocytometer (SystemBükerTürk; Incyto, Cheonan, Korea) and replated in 150 mm culture dishes (1 × 10^5^ cells per dish). These processes were continued until the MSCs stopped proliferating. MSC proliferation was analyzed by determining the population doubling time  or PDT^21,22^.

### CFU-F assays

CFU-F number was determined as described in previous report^23,24^. We expand MSCs (P2, P4, P6 and P8) cultures to 70–80% confluency and harvest with trypsin-EDTA and counted using a C-Chip hemocytometer as described previously. A glass Pasteur pipette was flamed at its tip to reduce its diameter, and the cells were drawn through the narrowed pipette several times to ensure cell separation. Cells were diluted in culture medium, and plated at about 100 cells per 100 mm tissue culture dishes. After incubation for 10–14 days at 37 °C in 5% humidified CO_2_, and wash with phosphate buffered saline, without Ca^++^ or Mg^++^, pH7.4 (PBS, Gibco), we stain cells with 1% (w/v) Crystal Violet solution (sigma-Aldrish, St. Louis, MO) in methanol for 5–10 min at room temperature. Cells were washed with PBS twice and the percentage of colonies with ≥3 mm diameter among total visible colonies was calculated.

### Ethics

All procedures and methods involving human samples were in accordance with approved guidelines. We received informed consents from all of the participants. This study was approved by the Institutional Review Board of the Catholic University of Korea (KC15TESE0026).

### Immunophenotyping

Immunophnotypes were evaluated using mouse anti-human monoclonal antibodies: fluorescein isothiocyanate (FITC)-conjugated HLA-DR (BD Biosciences, San Jose, CA) and Phycoerythin (PE)-conjugated CD73, CD90, CD105, CD11b, CD34, CD45 and CD79a. As a control, isotype PE-conjugated IgG1 and FITC-conjugated IgG2a (BD Biosciences) were used. The cell suspension containing 1 × 10^6^ cells was incubated with monoclonal antibodies for 15 minutes at room temperature in the dark and fixed with BD Cytofix^TM^ (BD Biosciences). The analysis was performed on FACSCalibur cytometer (BD Biosciences) and the resulting data were processed using CellQuest^TM^ Proversion 6.0 software (BD Biosciences).

### MSC-specific stemness gene expression

RNA was extracted using the RNeasy® Mini Kit (Qiagen, Hilden, Germany) according to the manufacturer’s instructions. cDNA was synthesized using Transcriptor First strand cDNA synthesis Kit (Roche, Mannheim, Germany). In order to confirm stemness nature, RT-PCR was performed with specific primers including *OCT4*, *SOX2*, *NANOG*, *GATA4*, *REX1*, *FGF4*, *SCF*, *TERT* and *HLA-ABC*. *GAPDH* was used for internal control. PCR conditions were 5 minutes at 96 °C, 35 cycles of 30 seconds at 96 °C, 30 seconds at 58 °C, 30 seconds at 72 °C and 5 minutes at 72 °C. PCR products were electrophoresed on 2% (w/v) agarose gels with GelRed Nucleic Acid Stain (Biotium, Hayward, CA, USA) and bands were observed under ultraviolet light.

### *In vitro* mesodermal differentiation

Cultured MSCs (1 × 10^5^) were dispensed in 6-well tissue culture plates (Nunc, Shanghai, China). When MSCs reached 70–80% confluency, the medium was replaced to differentiation media and incubated at 37 °C with 5% CO_2_ for 3 weeks. Adipogenic differentiation was induced using StemPro® adipogenesis differentiation kit (Gibco) and stained using Oil Red-O (sigma-Aldrish, St. Louis, MO). For chondrogenic differentiation, 2.5 × 10^5^ cells were centrifuged at 150 x g for 5 minutes in a 15 ml conical tube (BD Biosciences), then cultured with chondrogenesis differentiation kit (Gibco). Cell pellets were fixed with 4% (w/v) paraformaldehyde and embedded in paraffin. Sections were stained using alcian blue staining kit (ScienCell) and safranin O staining solution (Sigma-Aldrish, St. Louis, MO). Osteogenesis was induced by osteogenesis differentiation kit (Gibco) and stained with 2% (w/v) Alizarin Red Solution (ScienCell, Calsbad, CA).

RNA was extracted from differentiated MSCs as described above. qRT-PCR was performed with mesodermal differentiation specific gene primers including *ADPQR*, *LPL*, *PPARG*, *FABP4*, *SOX9*, *COL2A1*, *COMP*, *OPN*, *SP7* and *ALP*, and PowerUp^™^ SYBR^®^ green master mix (Applied Biosystems, Foster City, CA, USA).The primers sequences are presented in Supplementary Table S5. PCR reactions were run on a ViiA™ 7 Real-Time PCR system (Applied Biosystems) under standard conditions. After PCR, a dissociation curve was constructed in the range of 60 °C to 95 °C. The gene expression was normalized with a reference gene, glyceraldehyde-3-phosphate dehydrogenase (*GAPDH*) and undifferentiated MSCs were used as control. All experiments were repeated three times to test reproducibility.

### qPCR for measuring telomere length

To evaluate the shortening telomere during passaging, MSC telomere length was measured with a qPCR-based technique that compares telomere repeat sequence copy number to single-copy gene (36B4) copy number according to a previous study^14,25^. The telomere-specific primers (forward: 5′GGTTTGTTTGGGTTTGGGTTTGGGTTTGGGTTTGGGTT3′; reverse: 5′GGCTTGCCTTACCCTTACCCTTACCCTTACCCTTACCCT3′) and the 36b4 primers (forward: 5′CAGCAAGTGGGAAGGTGTAATCC3′; reverse: 5′CCCATTCTATCATCAACGGGTACAA3′ were prepared. All PCRs were performed on the Rotor-Gene Q real-time instrument (QIAGEN, Hilden, Germany). The thermal cycling profile for both amplicons began with 95 °C incubation for 10 min. For telomere PCR, there followed 25 cycles of 95 °C for 15 s, 58 °C for 1 min. For 36B4 PCR, there followed 30 cycles of 95 °C for 15 s, 58 °C for 1 min. Rotor-Gene Q software 2.0.2 was then used to generate the standard curve for each plate and to determine the dilution factors of standards corresponding to the T and S amounts in each sample. Relative T/S ratios will reflect relative length differences in telomeric DNA.

### Whole genome sequencing

For genomic DNA extraction, we used the DNeasy Blood & Tissue Kit (Qiagen, Germany) according to the manufacturer’s recommendations. Genomic DNA was obtained from different passages (P1, P3, P5, P7, and P9) of two MSC lines as well as from peripheral blood mononuclear cells from the same individuals as matched controls. The genomic library for whole genome sequencing was prepared according to the manufacturer’s instructions. Whole genome sequencing was performed using an Illumina ×10 platform according to the manufacturer’s recommendations (Illumina, USA) to obtain paired-end 100 bp sequencing reads. Whole genome sequencing data are available in Supplementary Table S1.

### Processing of sequencing data

Raw sequencing reads were aligned onto the reference human genome (hg19) using Burrows-Wheeler aligners (BWA version 0.7.13) with the default option^26^. Local realignment and score recalibration of the sequencing reads were performed using the Genome Analysis ToolKit (GATK)^27^. We used Picard and Samtools to process and manage the intermediate sequencing files during the analysis^28,29^.

### Mutation profiling

To identify somatic SNVs, we compared the MSCs harvested during *ex vivo* expansion (P1 to P9) to their parental MSCs using whole genome sequencing. Given the moderate sequencing coverage (mean, 30X), we used two SNV discovery algorithms (MuTect2 in GATK 3.5 and VarScan2^30^) and defined the overlap between the two algorithms as high-confidence SNVs (referred to as SNVs). Similarly, high-confidence indels were defined as the overlap of indels called by both MuTect2 and VarScan2. ANNOVAR was used to locate the somatic mutations onto known coding genes and to annotate their functional consequences^31^.

### Copy number profiling

For CNA profiling from whole genome sequencing, we used the VarScan2 algorithm^30^. The whole genome sequencing read depth differences between the MSCs and their matched normal controls whole genome were calculated across genomic bins. GC-corrected read depth was log_2_ transformed and median centered. For smoothing and segmentation of genomic bins, we used a circular binary segmentation algorithm^32^. As complementary platforms to obtain high-confidence CNAs, we also used the Affymetrix SNP6.0 Genotype Array. We used PennCNV algorithm to call CNAs from microarray genotype platforms^33^. We considered the CNAs identified by whole genome sequencing that are also supported by microarray-based genotyping platforms as high-confidence CNAs and used for the subsequent analyses.

## Electronic supplementary material

Supplementary Tables S1-S5
